# Web-Based Intervention (SunnysideFlex) to Promote Resilience to Posttraumatic Stress Disorder Symptoms During Pregnancy: Development and Pilot Study

**DOI:** 10.2196/53744

**Published:** 2024-11-01

**Authors:** Katherine C Paltell, Jennifer Duffecy, Pauline M Maki, Shiva Edalatian Zakeri, Anka A Vujanovic, Erin C Berenz

**Affiliations:** 1 Department of Psychology University of Illinois at Chicago Chicago, IL United States; 2 Department of Psychiatry University of Illinois at Chicago Chicago, IL United States; 3 Departments of Psychology & Psychiatry University of Illinois at Chicago Chicago, IL United States; 4 Department of Psychological & Brain Sciences Texas A&M University College Station, TX United States

**Keywords:** trauma, posttraumatic stress disorder, pregnancy, perinatal, web-based intervention, stress, postpartum, depression, child health, treatment, behavioral therapy, SunnysideFlex

## Abstract

**Background:**

Approximately 4% to 8% of pregnant individuals meet the criteria for current posttraumatic stress disorder (PTSD), a known risk factor for a multitude of adverse maternal and child health outcomes. However, PTSD is rarely detected or treated in obstetric settings. Moreover, available prenatal PTSD treatments require in-person services that are often inaccessible due to barriers to care. Thus, web-based interventions offer great potential in extending PTSD treatment to high-risk pregnant individuals by providing affordable, accessible care. However, there are currently no web-based interventions designed specifically for the treatment of PTSD symptoms during pregnancy.

**Objective:**

This study aims to develop and pilot a 6-week, web-based, cognitive behavioral therapy intervention for PTSD, SunnysideFlex, in a sample of 10 pregnant women with current probable PTSD. Consistent with established guidelines for developing and testing novel interventions, the focus of this pilot study was to evaluate the initial feasibility and acceptability of the SunnysideFlex intervention and preintervention to postintervention changes in PTSD and depression symptoms. This approach will allow for early refinement and optimization of the SunnysideFlex intervention to increase the odds of success in a larger-scale clinical trial.

**Methods:**

The SunnysideFlex intervention adapted an existing web-based platform for postpartum depression, Sunnyside for Moms, to include revised, trauma-focused content. A total of 10 pregnant women in weeks 16 to 28 of their pregnancy who reported lifetime interpersonal trauma exposure (ie, sexual or physical assault) and with current probable PTSD (scores ≥33 per the PTSD checklist for DSM-5) were enrolled in the SunnysideFlex intervention. Assessments took place at baseline and 6 weeks (postintervention).

**Results:**

All participants were retained through the postintervention assessment period. Engagement was high; participants on average accessed 90% of their lessons, logged on to the platform at least weekly, and reported a generally positive user experience. Moreover, 80% (8/10) of participants demonstrated clinically meaningful reductions in PTSD symptoms from baseline to postintervention, and 50% (5/10) of participants no longer screened positive for probable PTSD at postintervention. Most (6/10, 60%) of the participants maintained subclinical depression symptoms from baseline to postintervention.

**Conclusions:**

Findings from this small pilot study indicate that SunnysideFlex may be a feasible and acceptable mechanism for delivering PTSD intervention to high-risk, trauma-exposed pregnant women who might otherwise not have opportunities for services. Larger-scale trials of the intervention are necessary to better understand the impact of SunnysideFlex on PTSD symptoms during pregnancy and the postpartum period.

## Introduction

### Background

The perinatal period, defined here as the period during pregnancy and up to 1 year post partum [1,2], is a time of heightened vulnerability to psychopathology. Perinatal affective distress is associated with profound maternal, fetal, infant, and familial consequences [3,4], and if left untreated, carries a substantial economic burden, particularly to health and social care [5,6]. Consequently, the identification and management of behavioral health concerns during the perinatal period has been increasingly recognized as a global health priority by the World Health Organization. Widespread attention has been placed on perinatal mood and anxiety disorders, with the prevention and treatment of postpartum depression being a primary area of research interest [3]. However, considerably less focus has been directed to other prevalent disorders that often co-occur with depression or anxiety, such as posttraumatic stress disorder (PTSD). Women are more than twice as likely as men to develop PTSD in their lifetime [7], with high-severity trauma exposure (eg, physical assault, sexual assault) resulting in the greatest risk for subsequent development of PTSD [8,9]. Approximately 4% to 8% of pregnant women meet the criteria for current PTSD, and higher rates are observed among those who are disadvantaged and belong to minority communities [10-12], who endorse elevated rates of high-severity trauma exposure and lifetime PTSD [13,14].

### Health Outcomes Associated With Perinatal PTSD

Perinatal PTSD poses a major public health concern, as it is associated with a multitude of adverse outcomes for the mother and child. PTSD during the gestational period is associated with an increased risk for poor pregnancy and birthing outcomes, including miscarriage, ectopic pregnancy, preterm labor, and low birth weight [15-19], as well as increased engagement in high-risk behaviors, such as drug and alcohol use, smoking, and poor use of prenatal care [20,21]. PTSD and gestational stress, more broadly, also affect the neurobiological development of the infant. Research has demonstrated that babies of stressed mothers have alterations in the hypothalamic-pituitary-adrenal axis functioning of basal cortisol [22,23] and stress reactivity [24,25], as well as differences in their functional and structural brain development [26,27]. Although research evaluating the clinical implications of these alterations is limited, emerging research suggests they may contribute to later difficulties for the child related to temperament, inattention, cognition, and mood and anxiety disorders [28-31]. Furthermore, PTSD also contributes to various postpartum challenges, including lower rates of breastfeeding, impaired maternal-infant bonding, and maternal and infant emotional dysregulation [15,32]. Notably, the adverse effects of these challenges have the potential to extend through childhood and beyond. For example, impaired postpartum bonding has been shown to be related to severe deficits in the socioemotional development of the child, hence increasing the child’s vulnerability to developing psychiatric disorders later in life [33,34].

### Identification and Treatment of PTSD During the Perinatal Period

Despite the high prevalence and deleterious effects of perinatal PTSD, the identification and treatment of PTSD in obstetric settings is uncommon, largely because of systemic limitations in obstetric clinics [35]. Specifically, although public health initiatives have sought to enhance obstetric screening for depressive and anxiety symptoms [36,37], knowledge and use of PTSD symptom assessment in perinatal populations is uncommon. Obstetric staff is often not trained to recognize symptoms that may be associated with PTSD (eg, fear of pelvic exams, difficulty with reduction of tobacco or other substance use, anxiety that appears disproportionate to presenting circumstances, etc) or equipped to support women who screen positive for psychiatric difficulties, and these challenges are amplified among clinics serving high-risk trauma-exposed populations [35,38,39]. Without appropriate obstetric screening measures for PTSD, detection and referrals for treatment are almost nonexistent, except for when domestic violence is disclosed [39]. In addition to restrictions in provider services, research suggests that most patients will not initiate or seek help for their PTSD symptoms during pregnancy [38]. Even more, pregnancy is often an exclusionary factor in PTSD treatment-outcome studies, further restricting opportunities for the assessment and treatment of perinatal PTSD [40]. Taken together, accessible mechanisms for the detection and treatment of PTSD during pregnancy are critically needed.

Importantly, the small number of studies that have evaluated PTSD intervention during the perinatal period have shown promising findings, including reductions in PTSD symptoms during pregnancy [41] and at postpartum [41-44], reductions in depression symptoms [41,42], increased use of prenatal care [42,45] and satisfaction with labor [42], and improvements in mother-infant bonding [42]. Most of these interventions have been psychosocial, with a focus on providing trauma-focused psychoeducation to high-risk women through the delivery of clinician-administered and self-guided learning modules [41-45]. Module content, although variable, primarily focused on skill development related to managing emotions, interpersonal relationships, and trauma reactions during pregnancy, through educational content, instructive stories (ie, vignettes), skill practice, and self-reflection. For some of these studies, elements of cognitive-behavioral theory (eg, problem-solving; [44,45]) and interpersonal therapy (IPT) [41] were used to inform module development, whereas the empirical basis for other studies was less clear [42,43]. These previous studies were not explicitly trauma-focused and were not firmly rooted in gold-standard treatment approaches for PTSD (ie, cognitive behavioral therapy [CBT]). A large body of literature supports trauma-focused CBT as an effective treatment for PTSD [46]. More recently, a small clinical trial (N=8) evaluating the prenatal implementation of narrative exposure therapy intervention for PTSD symptoms found clinically meaningful reductions in PTSD symptoms across almost all participants (n=7) [47]. Consistent with prior literature highlighting the safety of exposure-based interventions in perinatal populations [48,49], this study found narrative exposure therapy to be well-tolerated in the sample of pregnant women. Collectively, these findings suggest that access to adequate PTSD treatments may help to meaningfully reduce symptom burden for trauma-exposed pregnant women during a critical developmental period.

### Utility of Online Interventions in Perinatal Populations

An important limitation across these previous studies, in addition to the dearth of CBT-based intervention, is the reliance on in-person attendance for receiving services, which in turn contributed to difficulties in enrolling and retaining participants [44,46,50]. Indeed, substantial barriers to mental health care (eg, long waitlists, lack of transportation, inadequate childcare, inflexible work schedules, etc) preclude many pregnant women from accessing and using available mental health interventions [51], and such barriers are more severe for women from disadvantaged backgrounds [52-54]. Thus, accessible, evidence-based PTSD interventions designed to reduce barriers to treatment for the perinatal population are warranted.

Online interventions, particularly those integrating principles of CBT, offer great potential in extending PTSD treatment to pregnant women by providing affordable, accessible care that circumvents these barriers. These interventions typically include a combination of didactic material and interactive CBT tools to practice skills, such as cognitive restructuring or behavioral activation [55]. Prior research suggests that online, evidence-based PTSD interventions are effective in reducing PTSD symptoms in the general population and in veteran samples [56-59]. In addition, online perinatal mental health interventions targeting symptoms of depression, anxiety, and stress are effective in improving maternal mental health [60,61]. Furthermore, online interventions have been found to be feasible, acceptable, and accessible during pregnancy [55,61,62].

### Overview of the Sunnyside for Moms Platform

One CBT internet intervention of particular interest for use in addressing perinatal mental health concerns is the Sunnyside for Moms platform (ie, Sunnyside). This web-based, interactive intervention was created to overcome barriers that often preclude women from seeking care for perinatal depression. The infrastructure was developed through user-centered design focus groups and feedback provided by both providers and women receiving perinatal care. Sunnyside integrates principles of both CBT and IPT to provide an evidence-based intervention for mood and anxiety concerns during the perinatal period, with the primary goal of preventing postpartum depression. Broadly, the platform delivers brief learning modules that contain psychoeducation, skill instruction, and vignettes related to common pregnancy and postpartum issues. Basic skills (eg, behavioral activation, cognitive restructuring, etc) are introduced to manage mood and anxiety symptoms, and skills are reinforced through interactive tools. In 2015, the first, 8-week iteration of Sunnyside was piloted in a sample of 25 perinatal women with low to moderate depressive symptoms [61]. Prevalence estimates for postpartum depression in at-risk women in the absence of an intervention are 17% [63]; at 6 weeks postpartum, only 4% of the women enrolled in Sunnyside met the criteria for depression. In addition, more than two-thirds of the participants were retained through the completion of the intervention period [61]. The Sunnyside platform has undergone multiple adaptations to align with digital mental health recommendations (eg, shortening the intervention length from 8 to 6 weeks and reducing the number of learning modules), ensure a more inclusive and relevant user experience, and target specific clinical outcomes (eg, breastfeeding, treatment for depression, etc). However, to date, no evidence-based web intervention has been designed to address PTSD symptoms and related concerns during pregnancy and postpartum.

### Summary, Aims, and Hypotheses

Taken together, the current literature suggests that web-based interventions are a feasible and effective avenue for delivering mental health services among both trauma-exposed and perinatal populations. However, there are currently no web-based interventions designed specifically for the treatment of PTSD during pregnancy. Such interventions have the potential to substantially improve maternal and child health outcomes in high-risk, trauma-exposed women who may otherwise not have opportunities for services.

The objective of this study was to develop and pilot a prototype of a 6-week CBT web-based intervention for PTSD, hereafter referred to as SunnysideFlex, in a sample (N=10) of pregnant women with lifetime interpersonal trauma exposure (ie, sexual or physical assault) and current probable PTSD. SunnysideFlex is an adaptation of the original Sunnyside platform, an established and effective evidence-based, web-based intervention for postpartum depression [61]. Consistent with current guidelines for developing new interventions [64], this study focuses on 2 integral phases of intervention development and testing: (1) refinement (eg, establishing essential intervention components, assessing feasibility, acceptability, and tolerability), and (2) proof-of-concept (eg, testing the intervention in a small sample, determining whether the intervention can produce a clinically significant impact on outcomes of interest). This approach will allow for early refinement and optimization of the SunnysideFlex intervention to increase the odds of success in a larger-scale trial.

The first aim was to test the initial feasibility and acceptability of the SunnysideFlex intervention to inform intervention refinement and methodological considerations for a larger-scale trial of the intervention. To test the feasibility of the SunnysideFlex intervention, we evaluated (1) the proportion of participants who met eligibility and subsequently enrolled in SunnysideFlex, (2) intervention engagement and adherence, and (3) barriers to retention and engagement. Acceptability was tested by analyzing (1) quantitative ratings on SunnysideFlex acceptability measures and (2) responses to open-ended program feedback.

The second aim was to evaluate clinically meaningful and significant changes in PTSD and depression symptoms pre to post SunnysideFlex intervention. Clinically meaningful changes were assessed by determining (1) the number of participants who reported preintervention to postintervention PTSD symptom reductions of at least 10-points on the PTSD Checklist for DSM-5 (PCL-5; [65]) and (2) the number of participants at preintervention and postintervention with depression symptom scores below the clinical threshold for treatment referral (ie, Patient Health Questionnaire-9 [PHQ-9] total score<10; [66]). Reliable change indices (RCIs; [67]) were used to examine individual clinically significant changes in preintervention to postintervention PTSD and depression symptoms.

## Methods

### Participants

Participants in this study were 10 pregnant women recruited through online advertisements that were disseminated nationwide using digital recruitment platforms, including Research Match, Facebook (Meta Platforms, Inc), and university and women’s health mass listservs. Advertisements were also distributed via email to local and national women’s health centers. Individuals who responded to online advertisements completed a brief web-based screener through REDCap (Research Electronic Data Capture; Vanderbilt University) [68,69] to assess eligibility. Inclusion criteria were as follows: (1) pregnant and between 16-28 weeks gestation, (2) ≥18 years of age, (3) ability to complete procedures in English, (4) currently engaged in prenatal care with a health care provider (eg, obstetrician and gynecologist, nurse, midwife, etc), (5) access to a broadband internet connection, (6) a history of one or more interpersonal traumatic events meeting *Diagnostic and Statistical Manual of Mental Disorders, Fifth Edition* (*DSM-5*) criterion A for PTSD [70], and (7) a positive screen for probable PTSD (ie, PCL-5 score of ≥33 [71,72]). Current prenatal care was required to ensure participants had adequate access to obstetric support for monitoring of their physical and emotional well-being during the study period. Traumatic event exposure was required to be interpersonal in nature given that interpersonal traumas (eg, sexual assault and intimate partner violence) are disproportionately experienced by women and are related to the worst mental health outcomes when compared with other trauma types (eg, natural disasters and motor vehicle accidents [8,73,74]).

Interested individuals were excluded from participation if they were pregnant with multiples; self-reported a history of a psychotic disorder, bipolar disorder, dissociative disorder, or substance use disorder; or were currently receiving medication or psychotherapy to treat psychopathology. In addition, individuals who disclosed that they are currently in an abusive or unsafe relationship were excluded from participation. This exclusion criterion was added due to concerns about the appropriateness of SunnysideFlex learning modules (eg, using in vivo exposures for overcoming avoidance behaviors associated with PTSD) for women actively experiencing intimate partner violence. More broadly, the SunnysideFlex intervention was not designed to support women in actively unsafe relationships, and mechanisms were not in place to address acute safety concerns related to intimate partner violence, should they arise. Individuals excluded based on self-reporting a current (past-year) abusive relationship were provided with electronic referrals to domestic violence resources upon completion of the eligibility screener via the REDCap platform.

### Procedures

**Figure 1:**
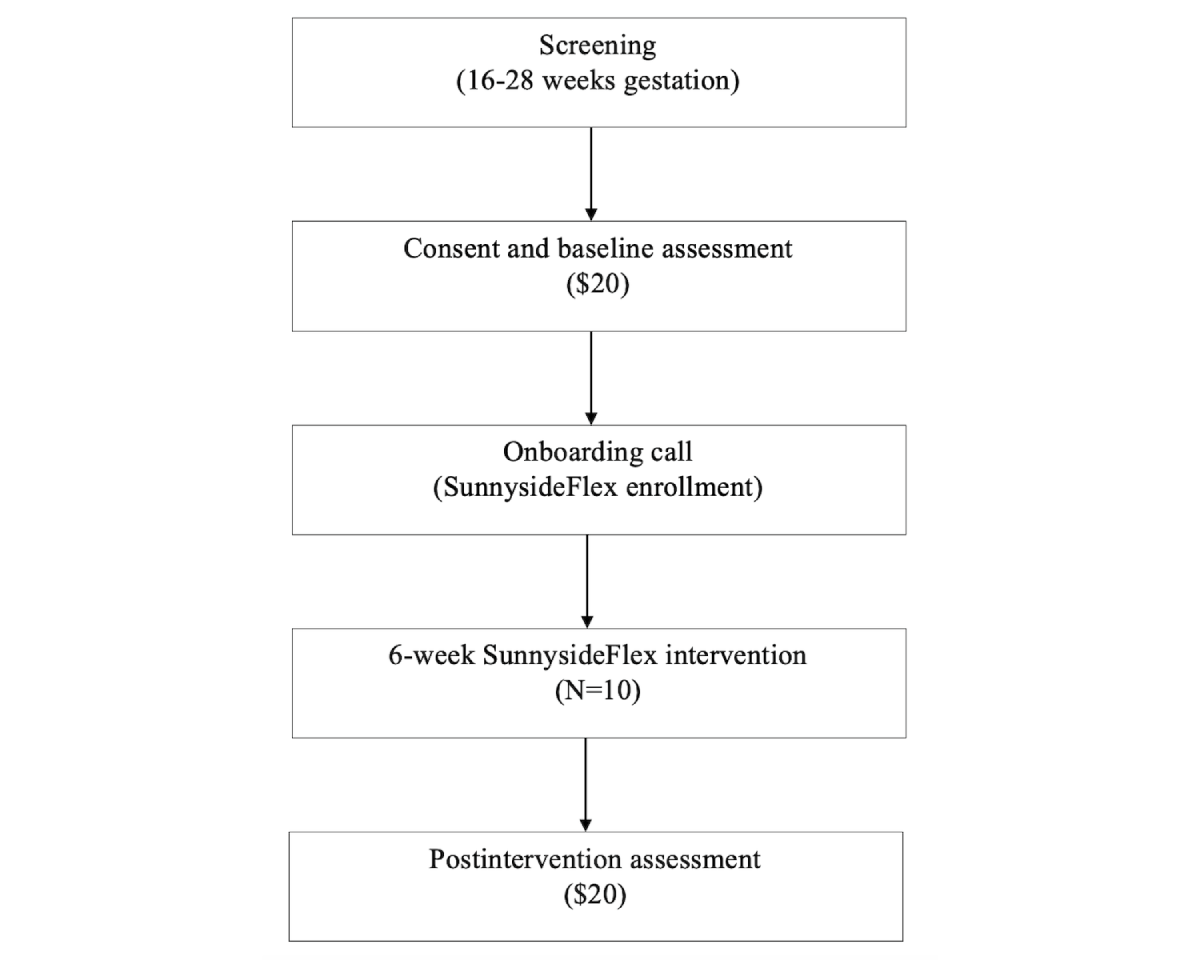
Study flowchart outlining key time points for a pilot study of a 6-week web-based intervention for posttraumatic stress disorder (PTSD) symptoms during pregnancy (SunnysideFlex) in a sample of pregnant women (N=10) with lifetime interpersonal trauma exposure and current probable PTSD.

Study design information is presented in Figure 1. Study data were collected and managed using REDCap electronic data capture tools [68,69]. If an individual qualified for enrollment based on the eligibility screener, they were automatically directed to complete an electronic consent (e-consent) instrument in REDCap. Eligible individuals who consented to participate in the study via the e-consent were immediately directed to a REDCap link to complete baseline assessment surveys. Baseline questionnaires assessed a variety of domains, such as demographic characteristics, medical and mental health history, social support, exposure to trauma and other stressful life events, current mood and anxiety symptoms, and trauma-related experiences.

Upon completing baseline questionnaires, eligible participants were immediately directed to schedule an initial onboarding phone call with a research assistant to review the components and expectations of the study and to provide an overview of the SunnysideFlex platform. This call was required to participate in the SunnysideFlex intervention and receive compensation. During the onboarding call, the research assistant reviewed participants’ responses to trauma-related screening items and provided brief psychoeducation about PTSD. Participants were also provided with their SunnysideFlex platform log-in information and taught how to access and use the platform. The 6-week intervention period began once the onboarding call was completed. Follow-up assessments related to depression and trauma symptoms, prenatal metrics, and satisfaction with the intervention were administered via REDCap on the completion of the 6 weeks of web-based SunnysideFlex lessons (postintervention).

Participants had the opportunity to opt-in to weekly text message queries from the research team, which were delivered to participants’ cell phones once every week during the 6-week intervention period (a total of 6 texts over the intervention period). These queries were standardized messages offering participants the opportunity to schedule a 15-minute support phone call with a member of the research team who is a master’s degree-level or above, to troubleshoot any technical difficulties and discuss skill use and engagement. Participants had the option to opt out of receiving text message queries at any point during the intervention period. Each participant also received text message reminders before their postintervention assessment due date.

### Ethical Considerations

Qualifying individuals provided electronic informed consent to participate at the time of enrollment. All procedures were approved by the institutional review board at the University of Illinois Chicago (approval number 2021-0002). Study data were deidentified to ensure privacy and confidentiality. Participants were compensated with a US $20 electronic Amazon gift card for each assessment time point (baseline and postintervention), for a total of up to US $40 in Amazon gift cards for study participation.

### Developing the SunnysideFlex Intervention

#### Overview

The SunnysideFlex intervention consists of 6 weeks of web-based lessons during pregnancy (12 intervention lessons plus 1 introductory lesson, a total of 13 lessons). SunnysideFlex is an adaptation of the established Sunnyside intervention, an interactive website with didactic material and interactive tools targeting skills to manage mood during and after pregnancy [61,75,76]. Refer to Multimedia Appendix 1 for an overview of intervention content.

#### Original Sunnyside Intervention

The original Sunnyside intervention website is based on CBT and IPT principles. Each lesson includes text and video material uniquely designed to provide information about pregnancy, postpartum issues, and components of CBT. The initial Sunnyside lesson introduces the cognitive-behavioral principles used throughout the intervention and explains how one’s thoughts and behaviors affect their mood and physical being. Subsequent lessons extend this information to cover a variety of prenatal and postpartum experiences, including common pregnancy worries; maintaining healthy interpersonal relationships and boundaries; and psychoeducation about physical exercise, body image, physical intimacy, breastfeeding, and employment issues during the perinatal period. Broadly, CBT skills (ie, cognitive restructuring, behavioral activation, and relaxation exercises) are taught for managing pregnancy stressors and life changes. At the conclusion of each lesson, participants are prompted with a Call to Action slide that encourages them to directly apply the CBT strategies that were learned in the lessons through 5 interactive tools: (1) thought restructuring (Think), (2) mood tracking (Feel), (3) activity scheduling and monitoring (Do), (4) relaxation (Relax), and (5) goal setting (Achieve). Associated tools serve to complement the lessons and directly apply to the CBT strategies that were discussed in the lessons. Participants are given unlimited access to the intervention and encouraged to use the site at least twice weekly as new modules become available every 3 to 4 days. Trauma-related content is not included in any of the Sunnyside lessons.

#### Adapting the Original Sunnyside Program

SunnysideFlex has adapted the original Sunnyside intervention to include revised, trauma-focused content that covers the impact of PTSD symptoms on the perinatal period and daily functioning. The intervention is intended for pregnant women who have previously experienced interpersonal trauma with associated current PTSD symptoms; lessons do not include content on pregnancy- and birth-related traumas. SunnysideFlex is consistent with current standards for CBT for PTSD [77,78] and is designed to be culturally relevant to pregnant women. SunnysideFlex introduces the same interactive Think, Feel, Relax, Do, and Achieve tools that are used in the original Sunnyside program; instructions for using these tools are tailored specifically to address trauma-related symptoms. Importantly, the SunnysideFlex program is identical to Sunnyside in its structure, such that there are 13 prenatal learning modules over a 6-week intervention period (12 intervention lessons plus one introductory lesson). During the 6-week intervention period, new lessons and accompanying tools are released twice a week, and participants receive an email notification upon the release of new material. Each lesson is self-guided and takes approximately 10 to 15 minutes to complete, consistent with the original Sunnyside intervention.

#### Overview of SunnysideFlex Content

SunnysideFlex modules covered the following areas: the first week of lessons (beginning at 16-28 weeks gestation) focused on psychoeducation related to trauma, PTSD, and the impact of PTSD on pregnancy and the postpartum period. Participants learned strategies (eg, deep breathing, progressive muscle relaxation, etc) to manage stress during pregnancy and were introduced to the Relax tool to encourage relaxation practice. The second week of lessons focused on cognitive experiences, such as common worries during pregnancy, with a special emphasis on unique concerns relevant to pregnant women with PTSD (eg, beliefs about the capacity to be a good parent, loss of control during labor and delivery, anxiety about physical exam procedures, etc). Participants learned about the impact of their thoughts on their feelings, and how trauma can impact thoughts people may have about themselves, others, and the world. The Think tool was introduced so that participants could track their thoughts. During the third week of lessons, participants learned strategies to challenge these thoughts and were encouraged to use the Think tool to practice cognitive restructuring. Week 3 content also provided psychoeducation about the impact of PTSD on behaviors and the role of behavioral activation in increasing engagement in pleasant activities. The Do tool was introduced to encourage women to track and schedule activities. The fourth week of lessons provided psychoeducation about the fight or flight response, the role of avoidance in maintaining PTSD symptoms, and the utility of in vivo exposures for overcoming avoidance behaviors. The Achieve tool encouraged participants to schedule in vivo exposure practice. In the fifth week of lessons, participants were introduced to trauma narrative writing. Lesson content also covered topics of social support, communication strategies, and establishing boundaries. Finally, the sixth and final week of content covered preparing for birth and summarized material from the past 6 weeks. Information to support participants, such as descriptions of evidenced-based PTSD treatments for individuals who wish to pursue additional treatment, was also provided in the final lesson.

### Measures

#### Screening Measures

##### Traumatic Life Events

Lifetime exposure to interpersonal traumatic events was assessed using the Traumatic Life Events Questionnaire **(**TLEQ; [79]), a comprehensive assessment of the range and count of potentially traumatic events. If one or more interpersonal traumatic events were endorsed on the TLEQ, respondents were then directed to indicate which of those events currently bothers them the most and respond to queries regarding whether the event was associated with a threat to one’s life, caused serious injury, or included sexual violence, consistent with *DSM-5* criterion A for PTSD.

##### PTSD Symptom Screening

Current symptoms of PTSD were assessed using the PCL-5 [65]. The PCL-5 is a well-validated screening tool for assessing PTSD symptoms and probable PTSD diagnosis and has been employed as the primary PTSD symptom measure in prior studies of pregnant samples [47]. The PCL-5 assesses the frequency of PTSD symptoms in the past 30 days in reference to the individual’s self-identified worst interpersonal traumatic event on the TLEQ (ie, the event perceived as most bothersome). A total score of 33 or greater represents a positive PTSD screen [69,70] and is the cutoff that was used in both this study and in previous studies evaluating PTSD during pregnancy [47].

##### Domestic Violence Screening

Current exposure to intimate partner violence was assessed using a subset of items from the Domestic Violence Initiative Screening Questionnaire [80]. This 4-item assessment asked the following questions with respect to the individual’s relationship with their current partner: (1) “Are you ever afraid of your partner?” (2) “In the last year, has your partner hit, kicked, punched, or otherwise hurt you?” (3) “In the last year, has your partner put you down, humiliated you, or tried to control what you can do?” and (4) “In the last year, has your partner threatened to hurt you?” Respondents were prompted to answer “yes,” “no,” or “not applicable, I am not currently in a relationship.” Respondents who answered “yes” to any of the 4 items were excluded from study participation; all ineligible respondents were provided with referral information to organizations geared toward supporting individuals in abusive or unsafe relationships.

#### Intervention Measures

##### Overview

There were 2 assessment time points (after eligibility was assessed and confirmed, and after the web-based e-consent was completed through REDCap): baseline and postintervention. Baseline assessments were obtained shortly after the participant completed the e-consent. Postintervention assessments were obtained at the conclusion of the 6-week SunnysideFlex intervention period (ie, 6 weeks after the onboarding call with the research team member). Participants completed web-based questionnaires via REDCap at both assessment time points to assess demographic information and outcomes of interest. The primary outcomes of interest in this study were intervention feasibility (ie, the proportion of participants who met eligibility and subsequently enrolled in the intervention, intervention engagement and adherence, and barriers to retention and engagement), intervention acceptability (ie, Likert ratings of intervention satisfaction and open-ended feedback about the SunnysideFlex program), and PTSD and depression symptom change. Relevant measures are as follows:

##### Demographics

This study-specific form assessed race, ethnicity, age, marital status, gender identity, sexual orientation, education, household size, number of children, pregnancy history, childcare, household income, and employment at baseline.

##### Health Services Utilization

The Health Services Utilization Questionnaire (modeled from a previous Sunnyside trial [75]) was used to assess participants’ use of medical, mental health, and breastfeeding services at baseline and postintervention.

##### PTSD Symptom Assessment

PTSD symptoms were assessed via the PCL-5 [65], described in more detail earlier under eligibility screener, at baseline and postintervention.

##### Depression Symptom Assessment

Broad depression symptoms were measured using the PHQ-9 [66] at baseline and postintervention. The PHQ-9 is a 9-item measure that uses a Likert scale to determine the frequency of experienced depressive symptoms over the past 2 weeks, with response options of “not at all” (0), “several days” (1), “more days than not” (2), and “nearly every day” (3). Scores of 5, 10, 15, and 20 on the PHQ-9 represent thresholds of mild, moderate, moderately severe, and severe depressive symptoms, respectively. The PHQ-9 has been well-studied and validated in prenatal populations [81,82].

##### Acceptability

Acceptability of the SunnysideFlex intervention was assessed using two measures: (1) the Usefulness, Satisfaction, and Ease of Use Questionnaire (USE; [83]); and (2) the SunnysideFlex Satisfaction Questionnaire (study-specific measure). The USE assessed participant ratings of overall intervention satisfaction, usefulness, ease of use, and ease of learning, and was the primary measure of intervention usability and satisfaction in previous Sunnyside pilot studies [61,75,76]. The USE instructs participants to respond to items on a Likert Scale ranging from “strongly disagree” (1) to “strongly agree” (7), with higher scores reflecting greater usability and satisfaction. Examples of items include “I am satisfied with [the SunnysideFlex program]” and “I would recommend [the SunnysideFlex program] to a friend.” The SunnysideFlex Satisfaction Questionnaire assessed participants’ program feedback in a variety of domains. Participants were asked to rate how well the program addressed their emotional needs, captured their trauma experiences and recovery, and fit with their racial, ethnic, and gender identity, on a scale ranging from “not at all” (1) to “very much so” (7). Participants were also invited to provide open-ended feedback about helpful and less helpful aspects of the program. Participants were then asked to identify the most and least helpful tools (Think, Feel, Do, Relax, and Achieve) and provide open-ended feedback about tool use. Finally, participants were asked to indicate how frequently they engaged in the in vivo and written exposure exercises and rate the helpfulness and tolerability of the exposures on a scale from “very unhelpful/I did not tolerate it well at all” (1) to “very helpful/I tolerated it very well” (7). This feedback was evaluated by the research team to inform future modifications of the intervention (postintervention).

##### Intervention Adherence Assessment

Adherence to the web-based SunnysideFlex intervention was measured by evaluating the number of logins to the intervention site during the 6-week prenatal intervention period, the number of lessons accessed, and engagement with the Think (thought record), Feel (mood rating), Do (activity scheduling and monitoring), Relax (relaxation), and Achieve (goal setting) platform tools. Data were used for feasibility analyses.

### Analytic Approach

#### Feasibility and Acceptability

Quantitative analyses were conducted in SPSS (version 28.0.1.0; IBM Corp). Feasibility was determined by examining *engagement rate*, defined as the proportion of individuals who met eligibility and subsequently enrolled in the intervention, and *intervention engagement and adherence*, including the number of logins during the intervention period, lessons accessed, and tools completed. Descriptive statistics were used to evaluate feasibility scores (ie, percentage of eligible participants who enrolled in the intervention, mean number of logins to the intervention platform, lessons read, and number of tools completed), as well as postintervention assessment completion and study attrition. *Barriers to retention and engagement* were also determined by evaluating text communication data with participants who had not accessed the platform for an extended period of time (ie, >1 week). Descriptive statistics were also used to evaluate acceptability, specifically ratings of intervention satisfaction, usefulness, and ease of use and learning on the USE and SunnysideFlex Satisfaction Questionnaire.

#### Symptom Outcomes

PTSD and depression scores from baseline to postintervention for each participant were evaluated to determine if the change was (1) clinically meaningful and (2) clinically significant. This approach is consistent with previous pilot work [47,84]. A 10-point reduction on the PCL-5 is considered the minimum reduction in the severity of symptoms to constitute clinically meaningful improvements in PTSD symptoms [67,85,86]. A PHQ-9 score of 10 is the clinical threshold for referral for treatment [66]. RCIs [67] were used to determine the statistical significance of symptom changes. The RCI statistic indicates the quantity and direction of symptom change for an individual, and whether that change is reliable and significant [67,87]. It is considered particularly useful in small samples due to its analysis of symptom change at an individual, rather than group level. It is also unique from other statistical methods (eg, *t* tests, ANOVAs, regression, etc), in that it considers the reliability of the measure used to evaluate symptom change.

The RCI is calculated using an individual’s preintervention and postintervention scores on a measure and the SE of the difference between those 2 scores (S_diff_). Specifically, 
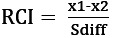
 , where x_1_ represents the individual’s preintervention score, x_2_ represents that same individual’s postintervention score, and S_diff_ describes the spread of the distribution of change scores that would be expected if no actual change occurred [67,87]. S_diff_ is calculated directly from the SE of the measurement (SE), as follows: 
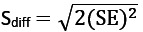
. The SE is calculated using the test-retest reliability of the measure (*r*_xx_) and the SD of the normal population for that measure (*s*_x_), such that 
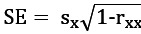
. When an RCI is greater than |1.96|, it is considered statistically reliable at the *P*<.05 level. In other words, it is unlikely that the postintervention score is not reflecting real change [67,87]. In this study, RCIs were calculated for participants’ preintervention and postintervention scores on the PCL-5 and PHQ-9. For the PCL-5, *r*_xx_=0.82 and *s*_x_=14 [69]. For the PHQ-9, *r*_xx_=0.84 and *s*_x_=3.8 [66].

## Results

### Screening

A total of 133 individuals completed the online eligibility screener over a 6-week period (October 24, 2022, to November 1, 2022, and January 6, 2023, to February 10, 2023). Recruitment and enrollment were paused from November 1, 2022, through January 6, 2023, to reduce the risk of study attrition during the holiday season. Most respondents accessed the screener through Research Match email invitations and Facebook advertisements. Approximately two-thirds of respondents identified as White, and one-quarter identified as Black/African American. Just over a quarter described their ethnicity as Hispanic/Latino. Of the respondents who completed the interpersonal trauma assessment portion of the screener (110/133, 82.7%), almost all endorsed lifetime exposure to at least one interpersonal traumatic event. The most frequently endorsed interpersonal traumas included unwanted sexual attention, witnessing family violence as a child, and experiencing physical abuse as a child. Approximately two-thirds of these respondents self-identified a “worst” interpersonal traumatic event that met criterion A for PTSD and completed the PCL-5 in relation to this event. The mean PCL-5 total score among these respondents was 49.27 (SD 15.99). Past-year intimate partner violence, the absence of lifetime interpersonal trauma meeting criterion A for PTSD, the absence of current prenatal care, and current mental health treatment (psychotherapy or pharmacological) were the most frequent reasons for ineligibility (Table 1).

Table 1 provides eligibility screener results (collected from a nationwide web-based recruitment effort) of a pilot study of a 6-week web-based intervention for PTSD symptoms during pregnancy (SunnysideFlex) in a sample of pregnant women with lifetime interpersonal trauma exposure and current probable PTSD.

**Figure 2:**
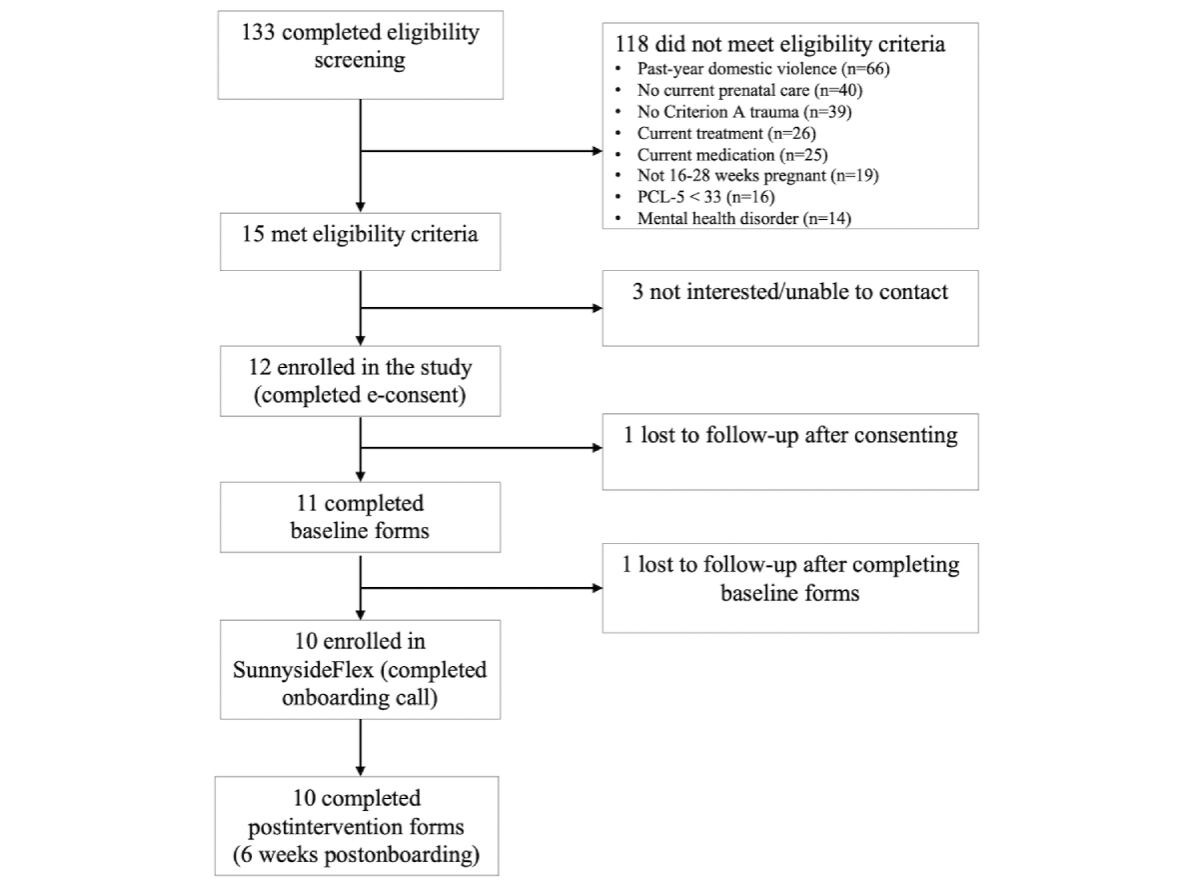
Participant flow CONSORT (Consolidated Standards of Reporting Trials) diagram for a pilot study of a 6-week web-based intervention (SunnysideFlex) for posttraumatic stress disorder (PTSD) symptoms during pregnancy in a sample of pregnant women (N=10) with lifetime interpersonal trauma exposure and current probable PTSD. Criterion A: Diagnostic and Statistical Manual of Mental Disorders, Fifth Edition criterion A for PTSD, defined as exposure to death, threatened death, actual or threatened serious injury, or actual or threatened sexual violence; PCL-5: PTSD Checklist for DSM-5, the primary measure of PTSD symptoms used in this study.

Overall, 15 (11.3%) of the 133 eligibility screener respondents met eligibility criteria and were invited to enroll in the study (ie, received links via email to complete the e-consent and baseline assessment measures). Three (20%) of these individuals did not enroll in the study and did not respond to contact attempts. One (7%) individual completed the e-consent and started their baseline assessment measures but did not complete these measures or proceed with the onboarding phone call (required to enroll in the SunnysideFlex intervention). One (7%) individual completed the e-consent and baseline assessment measures and scheduled their onboarding phone call but did not complete the call or engage in the SunnysideFlex intervention. Ultimately, 10 (67%) individuals completed the onboarding call and were enrolled in the SunnysideFlex intervention (Figure 2).

**Table 1:** Eligibility screener results.

			Values, n (%)		Values, mean (SD)
**Race (N=133)**					
	Black/African descent	30 (22.6)		—^a^	
	Central Asian	4 (3)		—	
	Native Hawaiian or Pacific Islander	1 (0.8)		—	
	South Asian, Southeast Asian, or East Asian	9 (6.8)		—	
	White	93 (69.9)		—	
**Ethnicity (N=133)**					
	Latino/Latina/Latinx or Hispanic	37 (27.8)		—	
	Not Latino/Latina/Latinx or Hispanic	96 (72.2)		—	
**Recruitment outlet (N=133)**					
	Research Match	78 (58.6)		—	
	Facebook	39 (29.3)		—	
	University mass emails	10 (7.5)		—	
	Other	6 (4.5)		—	
Currently pregnant (n=132)			122 (92.4)		—
**Gestational age (weeks; n=132)**					
	<16^b^	1 (0.8)		—	
	16-28	113 (85.6)		—	
	>28^b^	8 (6.0)		—	
Mean gestational age (n=132)			—		20.03 (2.88)
Multiples pregnancy^b^ (n=133)			16 (12.0)		—
Current prenatal care (N=133)			93 (69.9)		—
**Ever diagnosed with one of the following** ^b^ **(n=113)**					
	Psychotic disorder	12 (10.6)		—	
	Bipolar disorder	14 (12.4)		—	
	Dissociative disorder	9 (8.0)		—	
	Substance use disorder	13 (11.5)		—	
Currently taking medication for mental health^b^ (n=113)			25 (22.1)		—
Currently receiving mental health treatment^b^ (n=113)			26 (23.0)		—
**Intimate partner violence over the past year ^b^** **(n=113)**			66 (58.4)		—
	Afraid of current partner	45 (39.8)			
	Physically hurt by current partner	43 (38.1)			
	Controlled or humiliated by current partner	56 (49.6)			
	Threatened violence by current partner	49 (43.4)			
**Lifetime interpersonal trauma exposure^c^** **(n=110)**			99 (90)		—
	Uninvited or unwanted sexual attention	67 (60.9)			
	Witnessed family violence growing up	50 (45.5)			
	Physical abuse while growing up	49 (44.5)			
	Physical assault by intimate partner	48 (43.6)			
	Witnessed physical assault by stranger	47 (42.7)			
	Threatened death or serious injury	42 (38.2)			
	Sexual assault as an adult (≥18 years)	39 (35.5)			
	Childhood sexual assault before the age of 13 (someone ≥5 years)	37 (33.6)			
	Sexual assault as an adolescent (13-17 years)	37 (33.6)			
	Armed robbery	32 (29.1)			
	Physical assault by stranger	27 (24.5)			
	Childhood sexual assault before age 13 (someone close in age)	26 (23.6)			
Interpersonal trauma meeting criterion A^d^ for PTSD^e^ (n=110)			71 (64.54)		—
PCL-5^f^ total score ≥33 (n=71)			58 (81.7)		—
Mean PCL-5 total score (n=71)			—		49.27 (15.99)

^a^Not available.

^b^Denotes study exclusion criteria (if endorsed).

^c^Lifetime interpersonal trauma types assessed via Traumatic Life Events Questionnaire.

^d^Criterion A: Diagnostic and Statistical Manual of Mental Disorders, Fifth Edition criterion A for posttraumatic stress disorder, defined as exposure to death, threatened death, actual or threatened serious injury, or actual or threatened sexual violence.

^e^PTSD: posttraumatic stress disorder.

^f^PCL-5: PTSD checklist for DSM-5.

### Participants

Participants were 10 pregnant women in their second trimester of pregnancy with a median age of 34 years. The sample was predominantly White, not Hispanic or Latino, and married or in a committed relationship. Every participant attended at least some college, and almost all participants held a college or graduate degree. Most participants reported an annual household income of ≥US $51,000 and were employed full-time. The median household size was 2, and 4 (40%) participants reported having additional children. Five (50%) of the participants reported that their current pregnancy is their first pregnancy, and 3 (30%) participants reported previously experiencing a miscarriage. One (10%) participant reported preeclampsia in their current pregnancy; all other participants denied health or medical concerns associated with their current pregnancy at baseline (Table 2).

Table 2 provides baseline demographic characteristics of 10 pregnant women with lifetime interpersonal trauma exposure and current probable PTSD who enrolled in a small pilot study (N=10) evaluating a 6-week web-based intervention for PTSD symptoms during pregnancy (SunnysideFlex).

All participants endorsed a lifetime history of at least 1 interpersonal traumatic event on the TLEQ and met the criteria for a probable diagnosis of PTSD, as measured by a score of 33 or higher on the PTSD Checklist for DSM-5. Participants endorsed a median of 14.5 different types of lifetime traumatic events (including both interpersonal and noninterpersonal traumatic events). The most frequently endorsed interpersonal traumatic events were sexual assault as an adult, physical abuse by an intimate partner, and witnessing physical assault by an acquaintance or stranger. Of the 10 participants, 3 (30%) participants identified witnessing family violence during childhood as being their worst interpersonal traumatic event (ie, index trauma), and 2 (20%) participants identified unwanted sexual contact before age 13. The index traumas for the remaining participants included sexual assault as an adult, armed robbery, experiencing assault by an acquaintance or stranger, witnessing the assault of an acquaintance or stranger, and threatened death or serious harm. The median PCL-5 and PHQ-9 total scores at baseline were 55 and 8, respectively (Table 3).

Table 3 provides baseline trauma, PTSD symptom, and depression symptom characteristics of 10 pregnant women with lifetime interpersonal trauma exposure and current probable PTSD who enrolled in a small pilot study (N=10) evaluating a 6-week web-based intervention for PTSD symptoms during pregnancy (SunnysideFlex).

**Table 2:** Enrolled participants’ demographic characteristics (N=10).

			Participants, n (%)		Values, median (range)
Age (years)			—^a^		34 (22-41)
Estimated gestational age at screening (weeks)			—		20 (16-26)
**Race**					
	Black/African descent	1 (10)		—	
	White	7 (70)		—	
	South Asian or Southeast Asian	2 (20)		—	
**Ethnicity**					
	Latino/Latina/Latinx or Hispanic	1 (10)		—	
	Not Latino/Latina/Latinx or Hispanic	9 (90)		—	
**Relationship status**					
	Married	4 (40)		—	
	Committed relationship	4 (40)		—	
	Divorced	1 (10)		—	
	Single (never married)	1 (10)		—	
**Household size**			—		2 (2-5)
	First pregnancy	5 (50)		—	
	Number of participants with additional children	4 (40)		—	
	Number of additional children (if applicable)	—		1.5 (1-6)	
	Number of participants who previously miscarried	3 (30)		—	
	Number of miscarriages (if applicable)	—		1 (1-2)	
	Number of participants with current pregnancy complications	1 (10; preeclampsia)		—	
**Annual household income (US $)**					
	<15,000	1 (10)		—	
	15,000-20,999	2 (20)		—	
	51,000-75,999	2 (20)		—	
	76,000-100,999	2 (20)		—	
	101,000-149,999	2 (20)		—	
	≥150,000	1 (10)		—	
**Education**					
	Some college	1 (10)		—	
	2-year college degree (associate level)	1 (10)		—	
	4-year college degree (BA or BS)	4 (40)		—	
	Master’s degree	3 (30)		—	
	PhD degree	1 (10)		—	
**Occupation**					
	Homemaker	1 (10)		—	
	Unemployed	1 (10)		—	
	Employed part-time	1 (10)		—	
	Employed occasionally	1 (10)		—	
	Employed full time	6 (60)		—	

^a^Not appliable.

**Table 3:** Enrolled participants’ baseline trauma, posttraumatic stress disorder (PTSD), and depression characteristics (N=10).

		Participants, n (%)	Values, median (range)
**Interpersonal trauma types**			
	Uninvited or unwanted sexual attention	7 (70)	—^a^
	Sexual assault as an adult (≥18 years)	6 (60)	—
	Witnessed physical assault by stranger	5 (50)	—
	Physical assault by intimate partner	5 (50)	—
	Witnessed family violence growing up	4 (40)	—
	Threatened death or serious injury	4 (40)	—
	Physical abuse while growing up	4 (40)	—
	Sexual assault as an adolescent (13-17 y)	3 (30)	—
	Physical assault by stranger	3 (30)	—
	Childhood sexual assault before the age of 13 years (someone close in age)	3 (30)	—
	Childhood sexual assault before the age of 13 years (someone ≥5 years older)	3 (30)	—
	Armed robbery	3 (30)	—
**Noninterpersonal trauma types**			
	Unexpected death of a loved one	7 (70)	—
	Other accident	5 (50)	—
	Loved one surviving life-threatening illness, assault, or accident	4 (40)	—
	Abortion	4 (40)	—
	Natural disaster	3 (30)	—
	Motor vehicle accident	2 (20)	—
	Living, working, or military service in war zone	1 (10)	—
Total number of trauma types^b^		—	14.5 (3-23)
**Most distressing interpersonal traumatic event (criterion A)** ^c^			
	Witnessed family violence growing up	3 (30)	—
	Childhood sexual assault before age 13 (someone ≥5 years older)	2 (20)	—
	Sexual assault as an adult (18 or older)	2 (20)	—
	Armed robbery	1 (10)	—
	Physical assault by stranger	1 (10)	—
	Witnessed physical assault by stranger	1 (10)	—
	Threatened death or serious injury	1 (10)	—
PCL-5 total score^d^		—	55 (36-63)
PHQ-9 total score^e^		—	8 (3-18)
**PHQ-9 category for depressive symptoms**			
	None or minimal (0-4)	2 (20)	—
	Mild (5-9)	5 (50)	—
	Moderate (10-14)	1 (10)	—
	Severe (≥15)	2 (20)	—

^a^Not applicable.

^b^Number of lifetime trauma types assessed via Traumatic Life Events Questionnaire.

^c^Criterion A: Diagnostic and Statistical Manual of Mental Disorders, Fifth Edition criterion A for PTSD, defined as exposure to death, threatened death, actual or threatened serious injury, or actual or threatened sexual violence.

^d^PCL-5: PTSD Checklist for DSM-5, primary measure of PTSD symptoms used in this study.

^e^PHQ-9: Patient Health Questionnaire-9, the primary measure of depression symptoms used in this study.

### Intervention Feasibility

#### Engagement Rate and Attrition

A participant flow diagram (CONSORT [Consolidated Standards of Reporting Trials]) is presented in Figure 2. Overall, 10 (67%) of the 15 individuals who met the study eligibility criteria enrolled in the web-based SunnysideFlex intervention. Once enrolled in the intervention, 100% of participants were retained during the 6-week period and completed postintervention assessments.

#### Intervention Adherence

Participants logged in to the intervention platform an average of 10.20 times (SD 5.35) during the 6-week intervention period and accessed an average of 11.60 (SD 2.37) of the 13 available SunnysideFlex lessons. A total of 9 (90%) of 10 participants accessed at least 50% of the lessons, and 6 (60%) participants accessed 100% of them. On average, participants accessed a total number of 27.30 tools (SD 28.00). Almost all participants accessed the Think, Feel, and Achieve tools. The Do and Relax tools were each accessed by approximately 50% (5/10) of participants (Table 4).

Table 4 provides overall intervention adherence data (ie, engagement with the web-based intervention platform) of 10 pregnant women with lifetime interpersonal trauma exposure and current probable PTSD who enrolled in a small pilot study (N=10) evaluating a 6-week web-based intervention for PTSD symptoms during pregnancy (SunnysideFlex).

**Table 4:** Participant adherence data.

Program activity	Values, mean (SD; range)	Values, n (%)
Total logins	10.20 (5.35; 2-18)	—^a^
Lessons accessed^b^	11.60 (2.37; 6-13)	—
100% completion of lessons	—	6 (60)
50% completion of lessons	—	9 (90)
Tool: thought record (think; n=9)	4.50 (2.42; 0-8)	—
Tool: mood rating (feel; n=8)	6.60 (6.43; 0-22)	—
Tool: activity scheduling or monitoring record (do; n=4)	11.50 (18.09; 0-55)	—
Tool: relaxation (relax; n=5)	2.90 (4.51; 0-14)	—
Tool: goal setting (achieve; n=8)	1.80 (1.47; 0-5)	—
Total tools used	27.30 (28.00; 0-93)	—

^a^Not applicable.

^b^Participants were offered a total of 13 SunnysideFlex lessons during the 6-week intervention period.

#### Barriers to Adherence and Engagement

A total of 7 (70%) of 10 participants logged on to the platform at least weekly for the duration of the 6-week intervention period. Three (30%) of the participants logged on to the platform less than weekly. One of these participants shared that she was struggling to find time to access the platform due to unanticipated family difficulties and travel. Although she did not access the platform for the first 4 weeks of the intervention, she completed 9 of the 13 lessons and accessed 2 of the tools (Think, Achieve) over 2 sessions in the remaining 2 weeks of the intervention period. A second participant notified the team that she was traveling in a foreign country for 2 weeks and would complete her lessons upon returning home. She did not log on to the platform for a 3-week period but reengaged upon returning home, ultimately completing 11 (85%) lessons over 7 sessions and logging 19 tools. A third participant only accessed the platform 3 times during the intervention period. She completed a total of 6 (46%) lessons and did not log any tools. This participant did not provide a reason for her disengagement from the platform despite several prompts from the research team.

#### Text Message and Call Engagement

All 10 participants opted into receiving standardized weekly text message queries, which offered the chance to schedule a 15-minute support call with a SunnysideFlex team member to answer any questions they may have about their lessons or to discuss emotions that came up as they completed their lessons. In total, 60 text message queries (1 text/intervention week, for each participant) were sent to participants’ phones over the course of the study period. Only one phone call was requested and scheduled by a participant in response to a text message query. This participant was seeking clarification regarding study procedures (eg, postpartum lessons, compensation, etc) and was not experiencing emotional distress or technological difficulties, nor did they have questions about their lessons. Several of the participants often replied to the text messages providing an update on their progress. Examples of these replies included “Hi, thanks for the offer. The lessons have been very helpful and I will certainly spend more time on it,” and “It is going good right now I don’t have any questions.”

### Intervention Acceptability

#### Likert Ratings of Acceptability

Acceptability data, including USE subscale ratings and SunnysideFlex Satisfaction Questionnaire ratings, are presented in Table 5. Participants’ mean ratings on the USE, which uses a rating scale of 1 (strongly disagree) to 7 (strongly agree), were 5.00 (SD 1.11) for usefulness, 5.43 (SD 1.19) for ease of use, 6.10 (SD 1.05) for ease of learning, and 4.94 (SD 1.48) for overall satisfaction with the program. On the SunnysideFlex Satisfaction Questionnaire, which uses a scale from 1 (not at all) to 7 (very much so), participant’s average ratings were 5.60 (SD 1.35) for “Did you feel like the program addressed your emotional needs during pregnancy,” 5.60 (SD 1.35) for “Did you relate to how the program talked about trauma and recovery from trauma,” and 5.30 (SD 1.42) for “Did you feel the program fit with your experiences related to your race, ethnicity, and gender.”

#### In Vivo and Written Exposure Engagement

All participants reported practicing in vivo exposure exercises at least one time. Out of 10 participants, 8 (80%) practiced the written exposure exercises at least once. The 2 (20%) participants who did not practice written exposure provided several reasons for not practicing, including not having enough time, not understanding what to do, and not finding written exposure helpful. Average ratings for the helpfulness of exposure exercises in trauma recovery, on a scale from 1 (very unhelpful) to 7 (very helpful), were 5.14 (SD 1.07) for in vivo exposure and 5.17 (SD 1.47) for written exposure. Mean tolerability ratings, on a scale from 1 (not at all well tolerated) to 7 (very well tolerated) were 4.29 (SD 1.25) for in vivo exposure and 4.67 (SD 1.21) for written exposure, within the range of satisfactory (Table 5).

Table 5 provides intervention acceptability data of 10 pregnant women with lifetime interpersonal trauma exposure and current probable PTSD who enrolled in a small pilot study (N=10) evaluating a 6-week web-based intervention for PTSD symptoms during pregnancy (SunnysideFlex).

**Table 5:** Acceptability data.

Acceptability metric and item question			Values, mean (SD)		Values, n (%)
**USE^a^** **subscales**					
	Overall usefulness	5.00 (1.11)		—^b^	
	Overall ease of use	5.43 (1.19)		—	
	Overall ease of learning	6.10 (1.05)		—	
	Overall satisfaction	4.94 (1.48)		—	
**Program ratings^c^**					
	Did the program address your emotional health needs during pregnancy?	5.60 (1.35)		—	
	Did you relate to how the program talked about trauma and recovery from trauma?	5.60 (1.35)		—	
	Did the program fit with your experiences related to your race, ethnicity, and gender?	5.30 (1.42)		—	
**In vivo** **exposure ratings**					
	How much did you practice exposure exercises in real life? (Once or twice)	—		5 (50)	
	How much did you practice exposure exercises in real life? (3-4 times)	—		3 (30)	
	How much did you practice exposure exercises in real life? (more than 5 times)	—		2 (20)	
	How helpful were exposure exercises for your trauma recovery?	5.14 (1.07)		—	
	How well did you tolerate exposure exercises?	4.29 (1.25)		—	
**Written exposure ratings**					
	How much did you practice written exposure exercises? (once or twice)	—		2 (20)	
	How much did you practice written exposure exercises? (3-4 times)	—		5 (50)	
	How much did you practice written exposure exercises? (more than 5 times)	—		2 (20)	
	What are the main reasons you did not practice written exposure exercises? (Not enough time)	—		1 (10)	
	(If not at all) What are the main reasons you did not practice written exposure exercises? (I did not understand what to do)	—		1 (10)	
	(If not at all) What are the main reasons you did not practice written exposure exercises? (I did not think it would be helpful to do exposure)	—		1 (10)	
	How helpful were written exposure exercises for your trauma recovery?	5.17 (1.47)		—	
	How well did you tolerate written exposure exercises?	4.67 (1.21)		—	

^a^USE: Usefulness, Satisfaction, and Ease of Use Questionnaire [83], which uses a rating scale of 1 (strongly disagree) to 7 (strongly agree).

^b^Not applicable.

^c^All other rating items from the SunnysideFlex Satisfaction Questionnaire (study-specific measure). Rating scales for these items used a 1 to 7 scale, with higher numbers indicating greater satisfaction, helpfulness, and tolerability.

#### Tool Feedback

Participants were asked to identify the tools that they found most and least helpful. They were asked to check all tools that apply, and therefore multiple tools were indicated as most or least helpful for some participants. Participants overwhelmingly favored the Think tool, whereas the Achieve tool was less favored. Feedback about the Feel, Relax, and Do tools was variable. Participants also provided open-ended feedback about the tools. A common theme that emerged was dissatisfaction with the level of engagement and interaction provided by the tools. One participant wrote, “The Feel & Relax tools were pretty static & not as interactive as the others - they are where I would normally dive in, but I didn’t find the tools very engaging.” Another participant wrote, “I think the Achieve tool can do more, it should have a reminder sound to remind one of someone’s goal set*.* Participants also noted difficulties with remembering to use the tools. In addition, one participant noted that the Relax tool was broken; this was brought to the attention of the Sunnyside developer and the issue was promptly resolved (Table 6).

Table 6 presents feedback provided by 10 pregnant women with lifetime interpersonal trauma exposure and current probable PTSD who enrolled in a small pilot study (N=10) evaluating a 6-week web-based intervention for PTSD symptoms during pregnancy (SunnysideFlex). Feedback describes the perceived utility of 5 interactive tools within the web-based intervention (Think, Feel, Relax, Do, and Achieve).

**Table 6:** Participant responses to SunnysideFlex program tool feedback.

Which of the five Sunnyside tools did you find most helpful?		Which of the five Sunnyside tools did you find least helpful?	Please provide any additional feedback about the Think, Feel, Do, Relax, and Achieve tools here
**Participant 1**			
	Think	Feel	They were ỉn sequence. Helpful in every matter
**Participant 2**			
	Think	Relax	The Relax videos never worked
**Participant 3**			
	Think	Relax	—^a^
**Participant 4**			
	Feel	Do	I’m still struggling to use these often.
**Participant 5**			
	Think	Feel and relax	Feel & Relax tools were pretty static & not as interactive as the others - they are where I would normally dive in, but I didn’t find the tools very engaging
**Participant 6**			
	Think, do, and relax	Feel	I did like creating goals in achieve, but found it easy to forget about them
**Participant 7**			
	Think, feel do, relax	Achieve	I think the achieve tool can do more, it should have a reminder sound to remind one of someone’s goal set
**Participant 8**			
	Relax	Achieve	A lot of the tools didn’t work regularly so it became painful going in and out of the website and the reminders to do a lesson after I had already completed it.
**Participant 9**			
	Think, feel, and do	Achieve	I do wish that I received a direct link via email to a journal-like program that would make it a bit easier during days that I can’t carve the time out to login to the portal.
**Participant 10**			
	Think, feel, and relax	Think, feel, and relax	They help me evaluate my feelings and gave me relaxing technique.

^a^Not applicable.

#### Open-Ended Program Feedback

All participants answered free-response questions about what they liked and disliked about the SunnysideFlex program, and what changes they would recommend to improve the program experience. Responses to open-ended feedback items for each participant are presented verbatim in Table 7 and summarized here. Several themes emerged in these responses. First, participants expressed strong positive feelings about the instructive stories (ie, vignettes), which were provided to participants in both text and video format. One participant wrote, “I really liked the videos because they were able to help me feel heard and relate.” Another participant suggested that the program content include more videos and less reading. Two (20%) participants, however, expressed negative feelings toward the vignettes or videos, describing them as “generic” and “robotic.” Second, consistent with specific tool-related feedback (described earlier under Tool Feedback), many participants perceived the tools to be less helpful relative to other aspects of the program. Multiple participants described difficulties integrating tool use into their daily routine and program engagement. Nonetheless, some participants stated that specific tools exercises, including relaxation and goal setting, were particularly helpful. Another participant wrote that she appreciated the brief duration of the tool activities, which helped the program tasks feel more manageable. Finally, several participants recommended tailoring the program to individual trauma histories and needs. One (10%) participant suggested more personalized interaction from the research team, writing, “I really would like it if someone from the program reached out more directly to ask about my well-being” (Table 7).

**Table 7:** Participant responses to open-ended SunnysideFlex program feedback.

What parts of the program did you find to be the most helpful?		What parts of the program did you find less helpful?	What changes would make you feel like the program was a better fit for your experiences?	Do you have any other feedback on the program that you would like to share?
**Participant 1**				
	The lessons, they were thoughtful and made me understand myself and reflect better.	The Do part. But overall, it was effective and useful	Nothing	Nope!
**Participant 2**				
	Reading about how you can make changes	The additional exercises	More videos on similar scenarios	I really enjoyed reading the quotes that were added to the lesson plan
**Participant 3**				
	The real-life examples	Tracking	Easier to use	It was fine
**Participant 4**				
	I really liked the videos because they were able to help me feel heard and relate.	The tools are a little harder to get into the “habit” of using. I am trying to find a better way to navigate that.	Maybe a daily reminder text to do the tools, it’s so easy to become busy and caught up in day-to-day life or to just forget because pregnancy brain.	I think you guys are on the right track! Keep up the great work.
**Participant 5**				
	Videos & stories were good	The somatic healing practices were not very interactive - i read thru all of them at beginning but then did not have a way to weave in	Also include surviving hurricanes & natural disasters as an option for trauma	Getting e-mails so often felt like too much - but I liked how short the requested activities were - so felt more manageable – I’m still worried about the time commitment right after giving birth
**Participant 6**				
	I found the educational readings/videos to be helpful and clear, and I also liked the relaxation exercises	It’s not clear to me how often and how much one should be using the website, so I have not really been getting consistent readings on things like tracking my mood.	I think an app instead of a website would make it more easily accessible (I only log on when I am at work on my computer)	—^a^
**Participant 7**				
	The week 5 part one where it taught me how to confront my trauma and the week 6 part two where they left numbers and links on help on my PTSD	I think I find everything helpful	I don’t think I would change anything	No
**Participant 8**				
	Some of the anxiety exercises	The repetitiveness, the clocking my feelings every day.	More tailored to the needs of the person, i.e., different programs for dv survivors, rape survivors, child abuse survivors etc.	No
**Participant 9**				
	I really found the journaling exercises to be very helpful, and really helped me to reframe my thoughts.	I think the videos weren’t the most helpful because they were a little generic.	I really would like if someone from the program reached out more directly to ask about my wellbeing, and more interactive exercises.	No
**Participant 10**				
	The goals exercise	Some of the videos	Make more practical videos and less robotic	Have more videos and less reading

^a^Not applicable.

### PTSD and Depression Symptom Changes

Eight (80%) participants demonstrated clinically meaningful PTSD symptom improvements (ie, at least a 10-point reduction on the PLC-5). RCI analyses indicated that these reductions were clinically significant for four (40%) of the participants. In addition, 5 (50%) participants reported subthreshold PTSD symptoms (ie, <33 on the PCL-5) at postintervention.

Regarding depression symptoms, 2 (20%) participants reported clinically significant (per RCI analyses) reductions in depressive symptoms on the PHQ-9 from baseline to postintervention. In addition, 1 (10%) participant reported a clinically significant increase in PHQ-9 depression symptoms from baseline to postintervention. Post hoc analysis determined that the increase in symptoms was not due to an increase in somatic symptoms (PHQ-9 Somatic items) alone. At baseline, out of 10 participants, 7 (70%) participants endorsed depression symptoms below the clinical threshold for referral for treatment (ie, <10 on the PHQ-9); this number decreased to 6 (60%) participants at postintervention. Specifically, 6 participants who endorsed subthreshold PHQ-9 symptoms at baseline remained subthreshold at postintervention, whereas the 7th participant endorsed a clinically significant increase in her PHQ-9 score from baseline (total score=5) to postintervention (total score=15). This participant did not report any adverse events or new-onset pregnancy concerns during the 6-week perinatal period that could contribute to a worsening in depressive symptoms (eg, gestational diabetes). The 3 participants who were above the PHQ-9 clinical threshold at baseline remained above the threshold at postintervention. Two of these 3 participants also demonstrated subclinical (<10-point reductions) in PTSD symptoms on the PCL-5 (Table 8).

Table 8 provides data on individual PTSD and depression symptom changes (from baseline to postintervention) for 10 pregnant women with lifetime interpersonal trauma exposure and current probable PTSD who enrolled in a small pilot study (N=10) evaluating a 6-week web-based intervention for PTSD symptoms during pregnancy (SunnysideFlex).

**Table 8:** Individual posttraumatic stress disorder (PTSD) and depression symptom changes from baseline to postintervention.

Time point^a^		Gestational age (weeks)	PCL-5^b^	PHQ-9^c^
**Participant 1**				
	Baseline	26	44	3
	Postintervention	33	7 ^d,e^	3
**Participant 2**				
	Baseline	25	36	3
	Postintervention	33	20^e^	6
**Participant 3**				
	Baseline	24	56	8
	Postintervention	31	15^d,e^	2^d^
**Participant 4**				
	Baseline	22	59	8
	Postintervention	29	48^e^	7
**Participant 5**				
	Baseline	23	54	11
	Postintervention	30	36^d,e^	15
**Participant 6**				
	Baseline	19	39	5
	Postintervention	25	27^e^	9
**Participant 7**				
	Baseline	20	61	16
	Postintervention	27	58	10 ^d^
**Participant 8**				
	Baseline	16	61	18
	Postintervention	23	55	22
**Participant 9**				
	Baseline	17	51	9
	Postintervention	24	37^e^	9
**Participant 10**				
	Baseline	17	63	5
	Postintervention	24	32^d,e^	15^d^

^a^Postintervention assessment occurred approximately 6 weeks from baseline.

^b^PCL-5: PTSD Checklist for DSM-5 total raw score, the primary measure of PTSD symptoms used in this study.

^c^PHQ-9: Patient Health Questionnaire-9 total raw score, the primary measure of depression symptoms used in this study; PHQ-9 total score of ≥10 is the clinical threshold for referral for treatment.

^d^Reliable change index ≥|1.96|.

^e^Clinically meaningful symptom reduction on PCL-5 (ie, minimum 10-point reduction).

## Discussion

### Principal Findings

This study piloted a newly developed, 6-week web-based CBT intervention for PTSD (ie, SunnysideFlex) in a sample of 10 pregnant women with lifetime interpersonal trauma exposure and current probable PTSD. Consistent with established guidelines for developing and testing novel interventions [64], the focus of this pilot was to assess the following: (1) the initial feasibility and acceptability of the SunnysideFlex intervention and (2) preintervention to postintervention changes in PTSD and depression symptoms, to inform intervention refinement and methodological considerations for a pilot clinical trial. The preliminary results from this study are promising, in that pregnant women with a range of interpersonal trauma types and associated PTSD symptomatology were willing to engage with SunnysideFlex, reported a generally positive user experience, and demonstrated improvements in PTSD symptoms from baseline to postintervention.

### Aim 1: Feasibility and Acceptability Outcomes

The first aim of this pilot was to evaluate the initial feasibility and acceptability of the SunnysideFlex intervention. Results indicated that pregnant women with probable PTSD were willing to participate in a trauma-focused web-based intervention over a 6-week period. Most of the individuals (10/15, 67%) who met the eligibility criteria enrolled in SunnysideFlex, and 100% of enrolled participants were retained through postintervention assessments. Intervention engagement was also high; participants on average accessed 89% (11.60/15) of their lessons and logged on to the platform at least weekly. Although direct comparisons between SunnysideFlex and previous Sunnyside iterations are not possible due to differences in intervention length and module quantity, SunnysideFlex engagement statistics (eg, platform log-in, number of lessons accessed, tool usage, etc) do appear concordant with previous iterations [76] when adjusted for these differences. These results are highly encouraging given that existing web-based treatments for PTSD show an average dropout rate of 22% [57] and often require remote therapist support to enhance engagement and retention [59,88].

Engagement and retention also exceeded that of prior studies using in-person modalities to deliver prenatal trauma-focused interventions [41-43]. For example, in one pilot study, participants only completed an average of 3 out of the 5 in-person prenatal intervention sessions [41]. In a second study, almost 30% of participants dropped out before completing the first 4 of 10 prenatal learning modules [43]. These studies frequently cited barriers to care (eg, inadequate childcare, lack of transportation, inflexible work schedules, etc) as contributors to enrollment and retention difficulties. Thus, the high engagement with SunnysideFlex underscores the utility of web-based interventions in reducing barriers to treatment and suggests that this modality of intervention is appealing to trauma-exposed pregnant women. Two participants reported international travel and unanticipated familial stressors during the intervention period; however, they were able to reengage with the platform after sustained (ie, >2 weeks) periods of absence, highlighting the benefits yielded by the flexible nature of web-based interventions. A third participant was minimally engaged during the entire intervention period for unknown reasons, despite several prompts from the research team. These instances indicate the need in future trials to be more cognizant of potential barriers to engagement, perhaps through qualitative interviews with participants at preintervention and postintervention.

Program adherence may have in part been bolstered by the standardized weekly text message queries that were delivered to participants during the intervention period. These queries were intended to offer a phone call to participants in need of technological or content-specific support. However, they ultimately became a valuable means of communication with participants about program engagement and may have helped participants remember to access the platform. Specifically, only one participant opted to schedule a support call, but almost all participants responded to the queries to update the research team on their weekly lesson status. These responses provided additional information on barriers to engagement (ie, travel, familial stress, etc). Thus, future SunnysideFlex trials may consider modifying the function of the weekly text messages to focus explicitly on program progress and reminding participants to complete their lessons. The lack of participant interest in scheduling support phone calls may ultimately facilitate the upscaling of the intervention by reducing the number of clinically trained personnel required to manage the SunnysideFlex program.

Data on program usability, acceptability, and satisfaction suggested an overall satisfactory user experience. Participants also rated SunnysideFlex favorably in terms of meeting their emotional health needs, addressing their trauma experiences, and aligning with their individual identities. Open-ended feedback helped to shed light on program aspects that were less satisfactory, with the primary feedback being focused on the structure of the web intervention (eg, a desire for more interactive program components and user-friendly tools). Considering participants’ overwhelmingly positive feelings about the program’s instructive video stories (ie, vignettes), the desire for more interaction may in part be addressed by adding additional vignettes to future content iterations.

Importantly, participants found the in vivo and written exposure exercises to be both tolerable and helpful, consistent with prior studies demonstrating promising outcomes with online in vivo [58,89] and written [58] exposure in nonpregnant samples. The favorable ratings of in vivo and written exposure also complement existing literature on the acceptability of exposure-based interventions in perinatal populations [47-49]. Indeed, 2 participants identified the exposure exercises as being their favorite aspects of the program in their open-ended program feedback. Given that interventions with explicit exposures to trauma memories and triggers are more efficacious than nondirective treatments [90], participants’ positive feelings toward the exposure components are highly encouraging and suggest that there may be room to include additional exposure exercises in future SunnysideFlex trials. There appeared to be a slight preference for in vivo, as compared with written, exposure exercises. Whereas all participants reported practicing in vivo exposure at least once or twice, 2 participants did not practice written exposure due to not having enough time, not understanding what to do, and not finding written exposure helpful. It may be helpful to modify SunnysideFlex content (eg, adding additional videos, introducing interactive journaling exercises, etc) to increase comprehension of and motivation for the written exposure.

### Aim 2: PTSD and Depression Symptom Outcomes

The second aim of this pilot was to evaluate clinically meaningful and significant changes in PTSD and depression symptoms preintervention to post SunnysideFlex intervention. Results must be interpreted with caution due to the uncontrolled nature of the study. Nonetheless, it is promising that 80% (8/10) of the participants demonstrated clinically meaningful reductions in PTSD symptoms from baseline to postintervention. This finding is comparable to a small pilot study evaluating the prenatal implementation of narrative exposure therapy for PTSD, which found clinically meaningful reductions in PTSD symptoms in 7 of 8 participants [47]. In addition, 50% (5/10) of the current sample no longer screened positive for probable PTSD at postintervention. This is within the range of gold-standard PTSD interventions delivered to the general population, including those delivered in person [46] and web-based with remote therapist support [59,88].

Considering major depression co-occurs in approximately 50% of individuals with PTSD [91], the number of participants with clinically elevated depressive symptoms at baseline was relatively low (3/10, 30%). This may be in part due to the exclusionary criterion of past-year domestic violence, as this population experiences particularly high rates of comorbid PTSD and depression [92]. Encouragingly, almost all (6/7, 86%) participants with subclinical depressive symptoms at baseline remained below the threshold at postintervention. One participant showed a reliable worsening in depression symptoms from preintervention to postintervention, despite reliable and clinically meaningful improvements in her PTSD symptoms. Post hoc analysis determined that this increase was not due to increases in somatic depressive symptoms alone (eg, trouble sleeping, fatigue, etc), which can often overlap with normative experiences of pregnancy [93]. Although this increase could have been due to chance, depression outcomes will need to be explored further in subsequent SunnysideFlex studies. It is also important to note that 3 (30%) of 10 participants demonstrated a more severe baseline symptom presentation characterized by significant elevations in both PTSD and depression symptoms; 2 of these participants did not demonstrate clinically significant or reliable changes in PTSD or depression symptoms at postintervention. Thus, it may be that SunnysideFlex has a reduced clinical impact among individuals with more severe and comorbid symptom presentations. Postintervention exit interviews with participants may be particularly helpful for contextualizing symptom changes.

### Additional Considerations

The recruitment strategy through Facebook, Research Match, and university mass emails was adequate, averaging 22 respondents per week over a 6-week recruitment period. This rate was lower than that of a recent Sunnyside depression prevention trial [75], which averaged more than 100 weekly respondents through advertisements placed on a nationwide internet-based pregnancy application and forum. However, the base rate of perinatal PTSD (4%-8% [10-12]) is lower than that of perinatal depression (10%-20% [94]), and therefore enrollment rates must be considered in the context of the rarer perinatal PTSD phenotype. Nevertheless, larger-scale trials of the SunnysideFlex intervention should partner with similar applications to optimize recruitment outcomes.

It is noteworthy that 2 of the most common reasons for ineligibility in this study were domestic violence in the past year and the absence of prenatal care. These exclusion criteria were intended to ensure participant safety and adequate access to obstetric care, respectively. However, they resulted in the exclusion of many high-risk individuals who might have benefited from the SunnysideFlex intervention. The requirement of prenatal care may have also unintentionally biased the sample toward higher-income, more-resourced populations. Thus, future SunnysideFlex trials require revisions to enrollment criteria to better align with the intervention’s long-term goal of increasing access to perinatal PTSD intervention in a range of populations (eg, under-resourced communities with limited opportunities for mental health care, recent survivors of intimate partner violence, and racially and ethnically diverse individuals). Future trials may consider extending recruitment to include individuals without current prenatal care, while also providing support in connecting participants with prenatal medical services. Partnership with domestic violence organizations may also be warranted to provide tailored learning modules that adequately address the multifaceted nature of intimate partner violence, while also mitigating potential safety risks (eg, opportunities for safety planning, excluding in vivo exposure exercises from module content, etc). Shorter-term recruitment considerations also include adjusting the current domestic violence exclusion criteria timeframe from “past year” to “past 6 months” to expand access to SunnysideFlex while new content iterations are in progress.

In addition, SunnysideFlex was developed to support pregnant individuals who have previously experienced interpersonal trauma and does not encompass pregnancy and birth-related traumatic stress, such as medical complications or obstetric mistreatment (eg, physical harm during labor or delivery, nonconsensual medical procedures, etc). These trauma types warrant unique considerations for assessment and treatment and are beyond the scope of the current SunnysideFlex intervention. However, this remains an important line of work for future research, particularly given the high prevalence of self-reported traumatic birth experiences [95]. Future iterations of SunnysideFlex may consider including learning modules to target PTSD symptomatology associated with pregnancy and birth traumas.

### Limitations

First, the conclusions that can be drawn from this study are limited due to the small size of the pilot sample and the absence of a control condition. Thus, it cannot be determined whether SunnysideFlex was causally responsible for improvements in PTSD symptoms and maintenance of subclinical depression symptoms. Follow-up assessments were not completed, and therefore the postintervention course of symptoms is not known. Prior literature on the naturally occurring course of perinatal PTSD and depression symptoms offers insight into potential symptom trajectories. For example, Onoye et al [96] observed a declining trend in PTSD and depression symptoms across pregnancy and postpartum (early first trimester through 6 or more weeks postpartum), with a late third-trimester symptom “peak” followed by continued decline [96]. In addition, Muzik et al [97] found that pregnant women with high levels of PTSD symptoms at 28 weeks gestation showed substantial reductions in symptoms at postpartum. These reductions were hypothesized to be related to lower levels of perceived distress in the context of increased estradiol and progesterone concentrations [97]. Given the high severity of PTSD symptoms in the current SunnysideFlex sample, it is possible the observed downtrend in symptoms may continue into the postpartum period. However, prior research has also highlighted substantial variability in symptom course based on individual (eg, current or lifetime PTSD symptoms, demographic risk, etc) and environmental (eg, lack of social support, new trauma exposure, pregnancy-related stress, etc) factors, further underscoring the need for tailored interventions to address the dynamic nature of symptoms across gestational and postpartum periods. Relatedly, it is also not clear at this time which pregnant individuals with PTSD will benefit most from the SunnysideFlex program; larger-scale randomized trials of the intervention are needed to investigate possible predictors of outcomes.

Second, findings cannot be generalized to other pregnant populations or treatment settings. All participants were recruited via electronic recruitment outlets (Facebook and Research Match), and findings may not generalize to women recruited through other means (eg, primary care, obstetrics and gynecology clinics, etc). Similarly, the sample was predominately White and not Latino or Hispanic. Subsequent trials are needed to assess the acceptability of SunnysideFlex in more ethnically and racially diverse samples.

Finally, the primary measure of PTSD symptoms in this study, the PCL-5, assesses past-month symptomatology. Given that most participants completed postintervention forms 1 week after the conclusion of their lessons, this means that their reference point for PTSD symptom endorsement overlapped with the intervention period by approximately 3 weeks. The PCL-5 is a well-established tool for monitoring symptom change during and after treatment, and this method was consistent with other pilot studies of prenatal PTSD intervention [47]. However, future SunnysideFlex trials should incorporate past-week symptom assessments to capture symptoms outside of the 6-week intervention period.

### Conclusions

In conclusion, findings from this small pilot study offer initial support for the newly developed SunnysideFlex program for use in pregnant women with probable PTSD. Participants consistently used the intervention and retention was excellent, with 100% of participants completing postintervention assessments. Accordingly, participants were generally satisfied with the program user experience and provided valuable feedback to inform avenues for intervention refinement. Results were also encouraging with respect to reducing PTSD symptoms and maintaining subclinical depression symptoms, although they must be interpreted with caution due to the small group of participants. Overall, SunnysideFlex may be a feasible and acceptable mechanism for delivering PTSD intervention to high-risk, trauma-exposed pregnant women who may otherwise not have opportunities for services. Larger-scale randomized trials with extended follow-up periods are necessary to better understand the impact of the SunnysideFlex intervention on PTSD symptoms during pregnancy and the postpartum period.

## Appendix

Multimedia Appendix 1 SunnysideFlex Intervention Components (Adapted From Original Sunnyside Program).

## Abbreviations

CBT: cognitive behavioral therapy
CONSORT: Consolidated Standards of Reporting Trials
DSM-5: Diagnostic and Statistical Manual of Mental Disorders, Fifth Edition
IPT: interpersonal therapy
PCL-5: PTSD Checklist for DSM-5
PHQ-9: Patient Health Questionnaire-9
PTSD: posttraumatic stress disorder
RCI: reliable change index
TLEQ: Traumatic Life Events Questionnaire
USE: Usefulness, Satisfaction, and Ease of Use Questionnaire

## Data Availability

The datasets generated and analyzed during this study are available from the corresponding author on reasonable request.
