# Supplementary figures and images for: Web-Based Intervention (SunnysideFlex) to Promote Resilience to Posttraumatic Stress Disorder Symptoms During Pregnancy: Development and Pilot Study
Source: JMIR Form Res. 2024 Nov 1;8:e53744. doi: 10.2196/53744 (PMC11568404; doi:10.2196/53744)

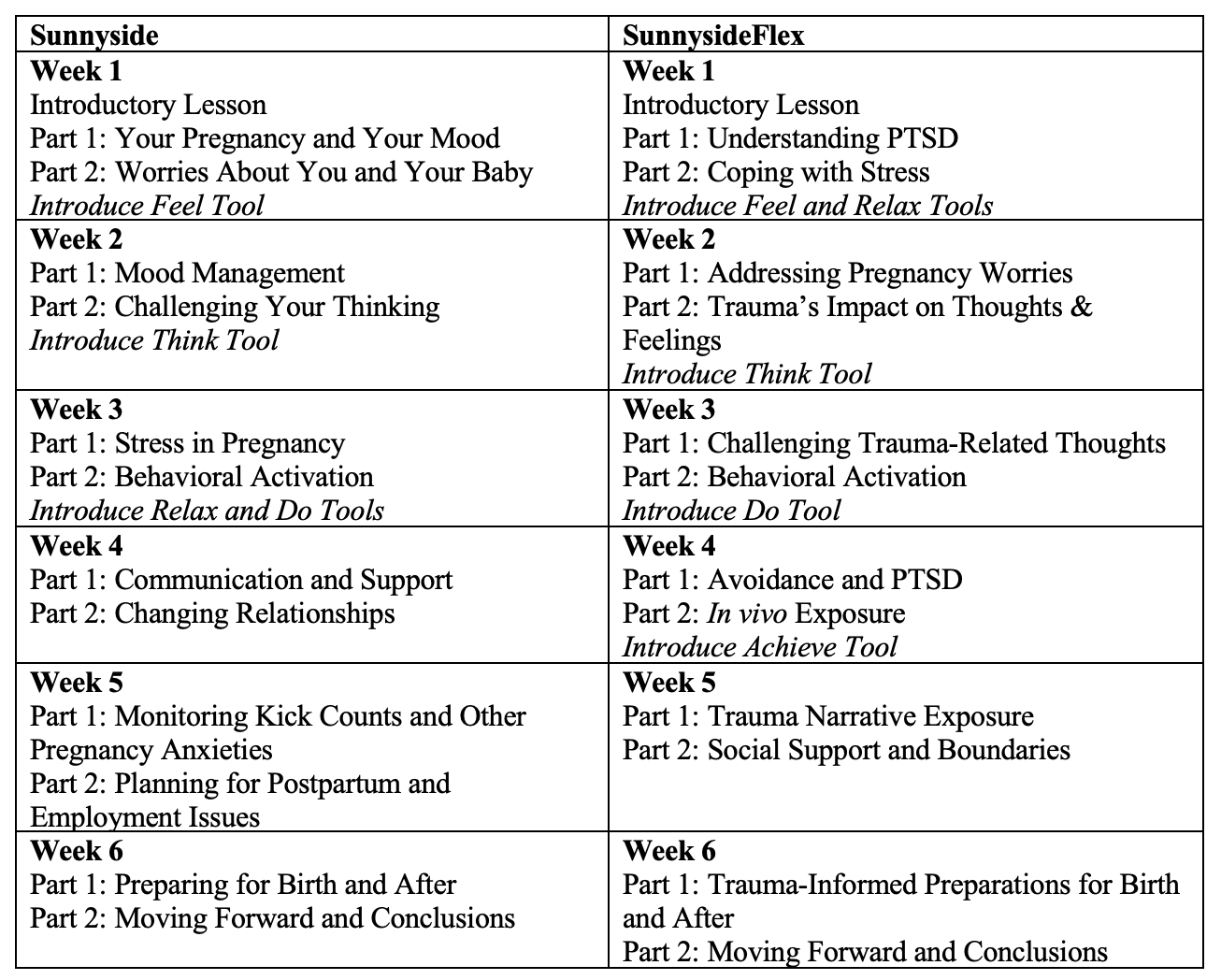

Supplement: Multimedia Appendix 1 [file formative_v8i1e53744_app1.png]
